# Supplementary material for: Force-field-enhanced neural network interactions: from local equivariant embedding to atom-in-molecule properties and long-range effects
Source: Chem Sci. 2023 Oct 3;14(44):12554–69. doi: 10.1039/d3sc02581k (PMC10646944; doi:10.1039/d3sc02581k)
Supplement: SC-014-D3SC02581K-s001 [file SC-014-D3SC02581K-s001.pdf]

Electronic Supplementary Material (ESI) for Chemical Science.
This journal is © The Royal Society of Chemistry 2023

Supporting information for :

Force-Field-Enhanced Neural Network Interactions: from Local Equivariant Embedding to Atom-in-Molecule properties and long-range effects

Thomas Plé,[1,*] Louis Lagardère,[1,†] and Jean-Philip Piquemal[1,‡]

[1] *Sorbonne Université, LCT, UMR 7616 CNRS, F-75005, Paris, France*

IMPACT OF LONG-RANGE DISPERSION INTERACTIONS

To highlight the importance of long-range interactions in the condensed phase, we investigated the impact of shortening the range of dispersion interactions on the structure of liquid water. Figure 1 shows the O-O radial distribution function of liquid water simulated using two versions of the FENNIX-OP1. The solid blue curve is the full version as shown in the main text of the article. The dashed orange curve is the same model with dispersion interactions smoothly cut off at 5.2Å, *i.e.* the same cutoff as for the local embedding (note that Coulomb interactions were unaltered and still long-range). We observe that neglecting the tail of the dispersion interactions results in a more structured liquid and slight shifts of the main peaks in the distribution toward shorter distances, in slightly worse agreement with experimental results than the original model.

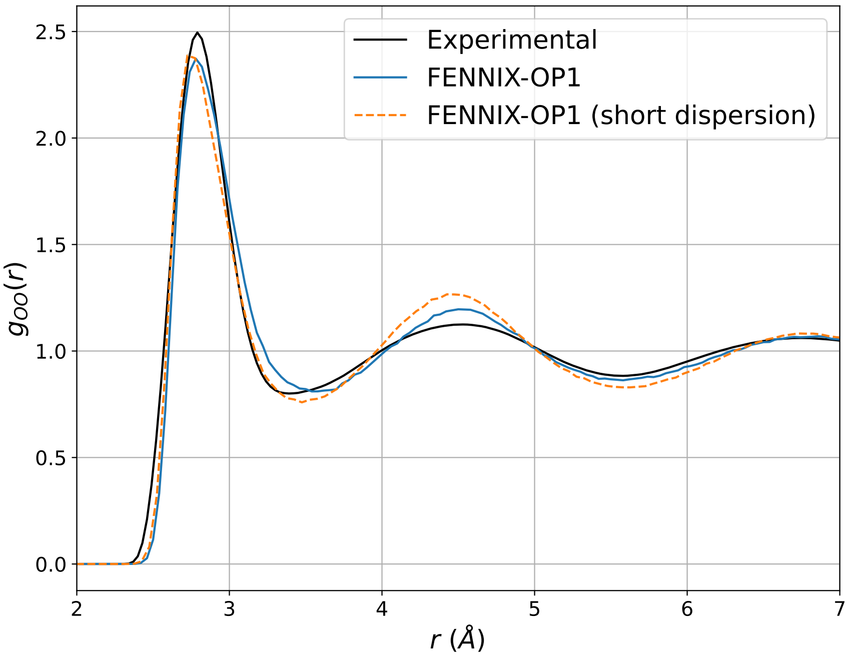

FIG. 1. O-O radial distribution of liquid water simulated using the full FENNIX-OP1 model (solid blue) and an altered model for which dispersion interactions are smoothly cut off at 5.2Å. Experimental results from [1]

* Corresponding author: thomas.ple@sorbonne-université.fr
† Corresponding author: louis.lagardere@sorbonne-université.fr
‡ Corresponding author: jean-philip.piquemal@sorbonne-université.fr

INFRARED SPECTRA OF LIQUID WATER

Figure 2 shows the infrared spectra of liquid water at 300K computed using the FENNIX-OP1 with adQTB and classical MD compared to experimental results from ref. [2]. IR spectra are computed from total dipole-derivative time-correlation functions estimated during a single Langevin MD trajectory. Effects of the Langevin thermostats are corrected using the deconvolution procedure described in ref. [3].

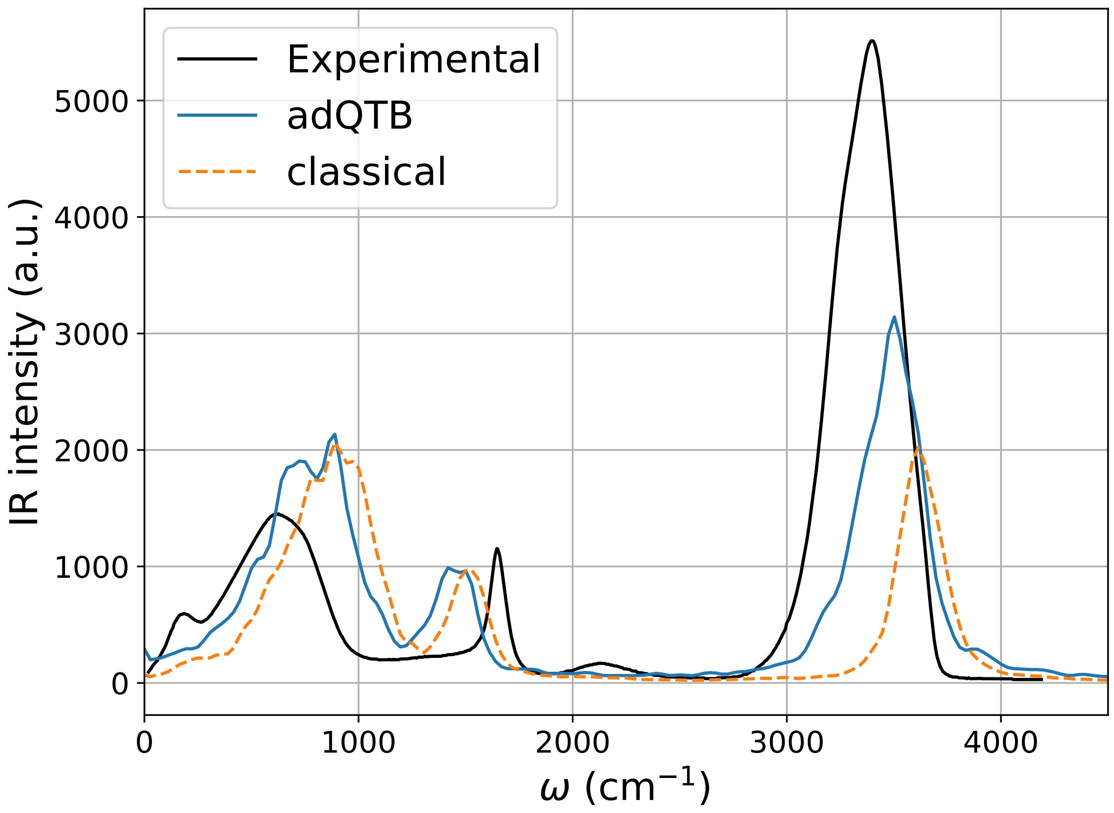

FIG. 2. Infrared spectrum of liquid water at 300K computed using the FENNIX-OP1 model with adQTB and classical MD. Experimental data from ref. [2]

---

[1] A. K. Soper, The radial distribution functions of water as derived from radiation total scattering experiments: Is there anything we can say for sure?, International Scholarly Research Notices

2013 (2013).

[2] J. E. Bertie and Z. Lan, Infrared intensities of liquids xx: The intensity of the oh stretching band of liquid water revisited, and the best current values of the optical constants of h2o (l) at 25 c between 15,000 and 1 cm- 1, Applied Spectroscopy **50**, 1047 (1996).

[3] M. Rossi, V. Kapil, and M. Ceriotti, Fine tuning classical and quantum molecular dynamics using a generalized Langevin equation, J. Chem. Phys. **148**, 102301 (2018), https://doi.org/10.1063/1.4990536.
